# Critical analysis of the relationship between arterial saturation and the ratio-of-ratios used in pulse oximetry

**DOI:** 10.1117/1.JBO.29.S3.S33313

**Published:** 2024-11-27

**Authors:** Giles Blaney, Angelo Sassaroli, Sergio Fantini

**Affiliations:** Tufts University, Department of Biomedical Engineering, Medford, Massachusetts, United States

**Keywords:** optical pulse oximetry, blood oxygen saturation, near-infrared spectroscopy

## Abstract

**Significance:**

Pulse oximetry is a well-established technique for the assessment of arterial oxygen Saturation (${\mathrm{SaO}}_{2}$). A quantitative analysis of its basic measurements may benefit its further development.

**Aim:**

We aim to derive and discuss the quantitative relationship between ${\mathrm{SaO}}_{2}$ and the measured quantity in pulse oximetry and the ratio-of-ratios (R) of pulsatile intensities at red and infrared wavelengths.

**Approach:**

The source of the pulsatile optical signal is the systolic and diastolic pulsation of the arterial compartment within the probed tissue. From a diffuse optics point of view, this is associated with an absorption change in the region that arterial blood expands into and a mean partial optical path length through that expanded arterial volume. This observation is the basis for a modified Beer–Lambert law analysis of the relationship between ${\mathrm{SaO}}_{2}$ and R.

**Results:**

The equation that relates ${\mathrm{SaO}}_{2}$ and R contains parameters that include the molar extinction coefficients of oxy- and deoxy-hemoglobin and the ratio of the mean partial optical path lengths in the arterial pulsatile expansion volume at the two wavelengths.

**Conclusion:**

The analysis reported here clarifies the source of the calibration equations used in pulse oximetry and provides guidance to assess the impact of various key optical parameters on the accuracy of pulse oximetry.

## 1 Background

Pulse oximetry is a well-established technique to estimate arterial oxygen saturation (${\mathrm{SaO}}_{2}$) from measurements of oxygen saturation (${\mathrm{SpO}}_{2}$) obtained from pulsatile optical signals at red (typically ∼660  nm) and infrared (IR) (typically ∼940  nm) wavelengths. The “p” in ${\mathrm{SpO}}_{2}$ indicates that its measurement is based on the arterial “pulse,” and it also points to the fact that it pertains to “peripheral” blood. Many helpful reviews have been written about pulse oximetry, including one that recounts its history,^1^ one written in a way to convey the engineering principles to medical practitioners,^2^ one that discusses the current technology and its limitations,^3^ and one that provides a summary of the basic principles and possible sources of error.^4^

Pulse oximeters operate in a diffuse optical regime. Another diffuse optical technique is near-infrared spectroscopy (NIRS), which is aimed more at bulk tissue properties and hemodynamics, as opposed to pulse oximetry, which specifically targets the arterial vascular compartment. The ability to perform absolute measurements with NIRS depends on the temporal domain employed: continuous-wave (CW),^5^ frequency-domain (FD),^6^ or time-domain (TD)^7^ (listed in order of increasing information provided about the investigated tissue). FD and TD are capable of absolute quantification of bulk tissue properties, whereas CW typically is only able to recover relative hemodynamics or oxygenation changes. CW NIRS methods are conceptually similar to those used in pulse oximetry. NIRS has been broadly applied to various human tissues, including the brain,^8^ skeletal muscle,^9^ and breast.^10^ Each application of NIRS centers around either the absolute optical properties of tissues or their dynamic changes, which are then linked to associated anatomical or physiological information. As pulse oximetry is entirely focused on the temporal dynamics of optical signals, we will only consider this type of application. In NIRS, temporal dynamics are analyzed with the modified the Beer–Lambert law (mBLL), which translates an intensity change (ΔI) into an absorption coefficient change ($\Delta \mu_{a}$) in tissue.^11^ At red and IR wavelengths, this $\Delta \mu_{a}$ typically results from a change in oxy-hemoglobin concentration ($\Delta [{\mathrm{HbO}}_{2}]$) and a change in deoxy-hemoglobin concentration (Δ[Hb]) in tissue, so that mBLL is used to retrieve hemodynamic information. Hemodynamic information is also what is sought in pulse oximetry, where $\Delta [{\mathrm{HbO}}_{2}]$ and Δ[Hb] result from a pulsatile arterial blood-volume change ($\Delta V^{\left(a\right)}$). This perspective serves to connect common data analysis methods in pulse oximetry to general NIRS concepts, which are formalized around the mBLL. In particular, we will discuss about possible interpretations of mBLL applied to pulse oximetry.

The philosophy of the methods used for pulse oximetry and NIRS is not the same. Nevertheless, pulse oximetry and NIRS share the same underlying physics, which relates to the propagation of light inside the tissue as considered by the mBLL. The physics tells us that the measured optical signal (the so-called ratio-of-ratios (R) in pulse oximetry) depends on the extinction coefficients (ε’s) of oxy-hemoglobin (${\mathrm{HbO}}_{2}$) and deoxy-hemoglobin (Hb) (i.e., $\varepsilon_{{\mathrm{HbO}}_{2}}$ and $\varepsilon_{\mathrm{Hb}}$) and the average partial optical path length in the pulsatile arterial blood volume ($\left\langle \ell_{\Delta V^{\left(a\right)}}\right\rangle$). Said in another way, $\left\langle \ell_{\Delta V^{\left(a\right)}}\right\rangle$ is the average path length that photons spend in the part of the arterial blood volume, which is expanded during the systolic phase ($\Delta V^{\left(a\right)}$) [in the text that follows, in Eq. (9), we will discuss an alternative interpretation based on the mBLL]. The way that these quantities are considered in relation to the measured optical signals is where pulse oximetry and NIRS differ. With pulse oximetry, a preliminary calibration is performed by fitting an equation (which may have various forms such as linear and quadratic) to the data of ${\mathrm{SaO}}_{2}$ versus ratio-of-ratios (R) collected on a calibration population of healthy subjects. By carrying out this exercise, one is in fact finding coefficients that relate to $\varepsilon_{{\mathrm{HbO}}_{2}}$, $\varepsilon_{\mathrm{Hb}}$, and $\left\langle \ell_{\Delta V^{\left(a\right)}}\right\rangle$ through empirical calibration. In Sec. 2, we derive an equation that relates ${\mathrm{SaO}}_{2}$ to R using the mBLL, similar to what was previously reported.^12^^,^^13^ Here, we provide a derivation of the equation and a critical description of key terms. We also point out how this equation shows the same functional dependence of ${\mathrm{SaO}}_{2}$ on R as the equations used for calibration in pulse oximetry, even though pulse oximetry equations may sometimes linearize the relationship between ${\mathrm{SaO}}_{2}$ and R. In all cases, the calibration equations used in pulse oximetry contain coefficients that depend on $\varepsilon_{{\mathrm{HbO}}_{2}}$, $\varepsilon_{\mathrm{Hb}}$, and $\left\langle \ell_{\Delta V^{\left(a\right)}}\right\rangle$. We hope that this derivation may be helpful to researchers in both fields of pulse oximetry and NIRS as it demonstrates the equivalence of the methods used in the two fields while also offering an opportunity to critically evaluate the meaning of the calibration coefficients used in pulse oximetry.

## 2 Derivation of the Relationship Between Arterial Oxygen Saturation (${\mathrm{SaO}}_{2}$) and Ratio-of-Ratios (R) Using the Modified Beer–Lambert Law (mBLL)

We start our derivation by expressing the amplitude of the effective absorption coefficient change ($\Delta \mu_{a}$) over the entire probed volume (V) in terms of the absorption coefficient of arterial blood ($\mu_{a}^{\left(a\right)}$) and pulsatile arterial blood-volume change ($\Delta V^{\left(a\right)}$), by using Beer’s law to relate $\mu_{a}^{\left(a\right)}$ to the oxy-hemoglobin concentration ($\left[{\mathrm{HbO}}_{2}\right]$) and the deoxy-hemoglobin concentration ([Hb]) in arterial blood ($[{\mathrm{HbO}}_{2}{]}^{\left(a\right)}$ and $[\mathrm{Hb}{]}^{\left(a\right)}$):

$$\Delta \mu_{a,\text{Red}}=\mu_{a,\text{Red}}^{\left(a\right)}\frac{\Delta V^{\left(a\right)}}{V}=(\varepsilon_{{\mathrm{HbO}}_{2},\text{Red}}\cdot [{\mathrm{HbO}}_{2}{]}^{\left(a\right)}+\varepsilon_{\mathrm{Hb},\text{Red}}\cdot [\mathrm{Hb}{]}^{\left(a\right)})\frac{\Delta V^{\left(a\right)}}{V},$$ (1)

$$\Delta \mu_{a,\mathrm{IR}}=\mu_{a,\mathrm{IR}}^{\left(a\right)}\frac{\Delta V^{\left(a\right)}}{V}=(\varepsilon_{{\mathrm{HbO}}_{2},\mathrm{IR}}\cdot [{\mathrm{HbO}}_{2}{]}^{\left(a\right)}+\varepsilon_{\mathrm{Hb},\mathrm{IR}}\cdot [\mathrm{Hb}{]}^{\left(a\right)})\frac{\Delta V^{\left(a\right)}}{V}.$$ (2)

Note that the subscripts represent either the red or IR wavelength, and the superscript (a) represents the arterial vascular compartment. We also observe that water absorption is neglected in Eqs. (1) and (2) for the sake of simplicity, but water absorption may be included after taking into account the water content in plasma^14^ and red blood cells.^15^ Solving the system of Eqs. (1) and (2) for $[{\mathrm{HbO}}_{2}{]}^{\left(a\right)}$ and $[\mathrm{Hb}{]}^{\left(a\right)}$, we get

$$[{\mathrm{HbO}}_{2}{]}^{\left(a\right)}=-\frac{\varepsilon_{\mathrm{Hb},\mathrm{IR}}\cdot \mu_{a,\text{Red}}^{\left(a\right)}-\varepsilon_{\mathrm{Hb},\text{Red}}\cdot \mu_{a,\mathrm{IR}}^{\left(a\right)}}{\varepsilon_{\mathrm{Hb},\text{Red}}\cdot \varepsilon_{{\mathrm{HbO}}_{2},\mathrm{IR}}-\varepsilon_{{\mathrm{HbO}}_{2},\text{Red}}\cdot \varepsilon_{\mathrm{Hb},\mathrm{IR}}},$$ (3)

$$[\mathrm{Hb}{]}^{\left(a\right)}=-\frac{-\varepsilon_{{\mathrm{HbO}}_{2},\mathrm{IR}}\cdot \mu_{a,\text{Red}}^{\left(a\right)}+\varepsilon_{{\mathrm{HbO}}_{2},\text{Red}}\cdot \mu_{a,\mathrm{IR}}^{\left(a\right)}}{\varepsilon_{\mathrm{Hb},\text{Red}}\cdot \varepsilon_{{\mathrm{HbO}}_{2},\mathrm{IR}}-\varepsilon_{{\mathrm{HbO}}_{2},\text{Red}}\cdot \varepsilon_{\mathrm{Hb},\mathrm{IR}}}.$$ (4)

Now, we write the definition of ${\mathrm{SaO}}_{2}$ as

$${\mathrm{SaO}}_{2}=\frac{[{\mathrm{HbO}}_{2}{]}^{\left(a\right)}}{[{\mathrm{HbO}}_{2}{]}^{\left(a\right)}+[\mathrm{Hb}{]}^{\left(a\right)}}.$$ (5)

Then, solving for ${\mathrm{SaO}}_{2}$ in terms of $\mu_{a,\text{Red}}^{\left(a\right)}$, $\mu_{a,\mathrm{IR}}^{\left(a\right)}$, and the ε’s yields

$${\mathrm{SaO}}_{2}=\frac{\varepsilon_{\mathrm{Hb},\mathrm{IR}}\cdot \mu_{a,\text{Red}}^{\left(a\right)}-\varepsilon_{\mathrm{Hb},\text{Red}}\cdot \mu_{a,\mathrm{IR}}^{\left(a\right)}}{(-\varepsilon_{{\mathrm{HbO}}_{2},\mathrm{IR}}+\varepsilon_{\mathrm{Hb},\mathrm{IR}})\mu_{a,\mathrm{Red}}^{\left(a\right)}+(\varepsilon_{{\mathrm{HbO}}_{2},\text{Red}}-\varepsilon_{\mathrm{Hb},\text{Red}})\mu_{a,\mathrm{IR}}^{\left(a\right)}}.$$ (6)

Dividing by $\mu_{a,\mathrm{IR}}^{\left(a\right)}$ gives:

$${\mathrm{SaO}}_{2}=\frac{\varepsilon_{\mathrm{Hb},\mathrm{IR}}(\frac{\mu_{a,\text{Red}}^{\left(a\right)}}{\mu_{a,\mathrm{IR}}^{\left(a\right)}})-\varepsilon_{\mathrm{Hb},\text{Red}}}{\left(-\varepsilon_{{\mathrm{HbO}}_{2},\mathrm{IR}}+\varepsilon_{\mathrm{Hb},\mathrm{IR}})(\frac{\mu_{a,\mathrm{Red}}^{\left(a\right)}}{\mu_{a,\mathrm{IR}}^{\left(a\right)}})+(\varepsilon_{{\mathrm{HbO}}_{2},\text{Red}}-\varepsilon_{\mathrm{Hb},\text{Red}}\right)}.$$ (7)

Now, we introduce the mBLL

$$\Delta \mu_{a,\text{Red}}=\mu_{a,\text{Red}}^{\left(a\right)}\frac{\Delta V^{\left(a\right)}}{V}=\frac{\ln(I_{0}/I{)}_{\text{Red}}}{{\left\langle \ell_{\Delta V^{\left(a\right)}}\right\rangle }_{\text{Red}}}≊-\frac{(\Delta I/I_{0}{)}_{\text{Red}}}{{\left\langle \ell_{\Delta V^{\left(a\right)}}\right\rangle }_{\text{Red}}},$$ (8)

$$\Delta \mu_{a,\mathrm{IR}}=\mu_{a,\mathrm{IR}}^{\left(a\right)}\frac{\Delta V^{\left(a\right)}}{V}=\frac{\ln(I_{0}/I{)}_{\mathrm{IR}}}{{\left\langle \ell_{\Delta V^{\left(a\right)}}\right\rangle }_{\mathrm{IR}}}≊-\frac{(\Delta I/I_{0}{)}_{\mathrm{IR}}}{{\left\langle \ell_{\Delta V^{\left(a\right)}}\right\rangle }_{\mathrm{IR}}},$$ (9)

where $\Delta I=I-I_{0}$ is the change of detected intensity between two states of the medium (a baseline state $I_{0}$ and a perturbed state I), and $\Delta V^{\left(a\right)}$ is the change of arterial blood volume between these two states. We point out that even though the choice of baseline and perturbed states is totally arbitrary, this choice has a consequence for the interpretation of $\left\langle \ell_{\Delta V^{\left(a\right)}}\right\rangle$. For example, if we choose the diastolic state as baseline (i.e., $\Delta V^{\left(a\right)}>0$), then $\left\langle \ell_{\Delta V^{\left(a\right)}}\right\rangle$ is the mean path length in those tissue regions where the arterioles are expanding into. On the contrary, if we choose the systolic state as baseline (i.e., $\Delta V^{\left(a\right)}<0$), then $\left\langle \ell_{\Delta V^{\left(a\right)}}\right\rangle$ is the mean path length in those tissue regions where the arterioles are receding from. In both cases, we expect that the optical properties of arterial blood (related to hematocrit and ${\mathrm{SaO}}_{2}$) are going to substantially affect $\left\langle \ell_{\Delta V^{\left(a\right)}}\right\rangle$ [but may have a lesser impact on the ratio of partial path lengths of Eq. (10)]. We can connect the $\Delta I/I_{0}$ of Eqs. (8) and (9) to typical measurands of pulse oximetry, if we choose $I_{0}$ as the detected intensity at an intermediate state between diastolic and systolic phases [so that it becomes the direct current (DC) component in pulse oximetry] and the ΔI as the amplitude change from pulsation (i.e., ΔI is the same as the alternating current (AC) component of pulse oximetry]. Finally, the approximate equality in Eqs. (8) and (9) holds if $\Delta I/I_{0}\ll 1$, which is the case we consider here, even though it was noted that this approximation may not always be accurate in pulse oximetry.^16^^,^^17^ Continuing the derivation, next we divide the two mBLL equations [Eqs. (8) and (9)] at the two wavelengths to yield

$$\frac{\mu_{a,\text{Red}}^{\left(a\right)}}{\mu_{a,\mathrm{IR}}^{\left(a\right)}}≊\frac{(\Delta I/I_{0}{)}_{\text{Red}}}{(\Delta I/I_{0}{)}_{\mathrm{IR}}}\frac{\langle \ell_{\Delta V^{\left(a\right)}}\rangle_{\mathrm{IR}}}{\langle \ell_{\Delta V^{\left(a\right)}}\rangle_{\text{Red}}}.$$ (10)

Here, it is important to note that $\Delta I/I_{0}$ is exactly what is called AC/DC (i.e., pulsatile amplitude divided by average signal) in the pulse oximetry literature. Given that the ratio-of-ratios (R) is defined as

$$R=\frac{(\mathrm{AC}/\mathrm{DC}{)}_{\text{Red}}}{(\mathrm{AC}/\mathrm{DC}{)}_{\mathrm{IR}}}=\frac{(\Delta I/I_{0}{)}_{\text{Red}}}{(\Delta I/I_{0}{)}_{\mathrm{IR}}},$$ (11)

we can rewrite Eq. (10) as

$$\frac{\mu_{a,\text{Red}}^{\left(a\right)}}{\mu_{a,\mathrm{IR}}^{\left(a\right)}}≊R\frac{\langle \ell_{\Delta V^{\left(a\right)}}\rangle_{\mathrm{IR}}}{\langle \ell_{\Delta V^{\left(a\right)}}\rangle_{\text{Red}}}.$$ (12)

Finally, we substitute Eq. (12) into Eq. (7) to get

$${\mathrm{SaO}}_{2}≊\frac{-\varepsilon_{\mathrm{Hb},\text{Red}}+\varepsilon_{\mathrm{Hb},\mathrm{IR}}(\frac{{\left\langle \ell_{\Delta V^{\left(a\right)}}\right\rangle }_{\mathrm{IR}}}{\langle \ell_{\Delta V^{\left(a\right)}}\rangle_{\text{Red}}})R}{(\varepsilon_{{\mathrm{HbO}}_{2},\text{Red}}-\varepsilon_{\mathrm{Hb},\text{Red}})+(-\varepsilon_{{\mathrm{HbO}}_{2},\mathrm{IR}}+\varepsilon_{\mathrm{Hb},\mathrm{IR}})(\frac{\langle \ell_{\Delta V^{\left(a\right)}}\rangle_{\mathrm{IR}}}{\langle \ell_{\Delta V^{\left(a\right)}}\rangle_{\text{Red}}})R}.$$ (13)

We observe that Eq. (13) is formally identical to previously reported equations for ${\mathrm{SaO}}_{2}$ as a function of R,^12^^,^^13^ with the key difference that Eq. (13) contains a ratio of partial optical path lengths ($\left\langle \ell_{\Delta V^{\left(a\right)}}\right\rangle$’s) rather than a ratio of effective total optical path lengths through the entire tissue. This difference is crucially important because the ratio $\langle \ell_{\Delta V^{\left(a\right)}}\rangle_{\mathrm{IR}}/\langle \ell_{\Delta V^{\left(a\right)}}\rangle_{\text{Red}}$ has a different value and features a different dependence on ${\mathrm{SaO}}_{2}$ and other anatomical and biological variables than the ratio of the total optical path lengths at IR and red wavelengths, as was reported before. To arrive at a compact version of the equation, we rewrite Eq. (13) as

$${\mathrm{SaO}}_{2}≊\frac{A+BR}{C+DR},$$ (14)

where

$$A=-\varepsilon_{\mathrm{Hb},\text{Red}},$$ (15)

$$B=\varepsilon_{\mathrm{Hb},\mathrm{IR}}\frac{\langle \ell_{\Delta V^{\left(a\right)}}\rangle_{\mathrm{IR}}}{\langle \ell_{\Delta V^{\left(a\right)}}\rangle_{\text{Red}}},$$ (16)

$$C=\varepsilon_{{\mathrm{HbO}}_{2},\text{Red}}-\varepsilon_{\mathrm{Hb},\text{Red}},$$ (17)

$$D=(-\varepsilon_{{\mathrm{HbO}}_{2},\mathrm{IR}}+\varepsilon_{\mathrm{Hb},\mathrm{IR}})\frac{\langle \ell_{\Delta V^{\left(a\right)}}\rangle_{\mathrm{IR}}}{{\left\langle \ell_{\Delta V^{\left(a\right)}}\right\rangle }_{\text{Red}}}.$$ (18)

**Fig. 1:**
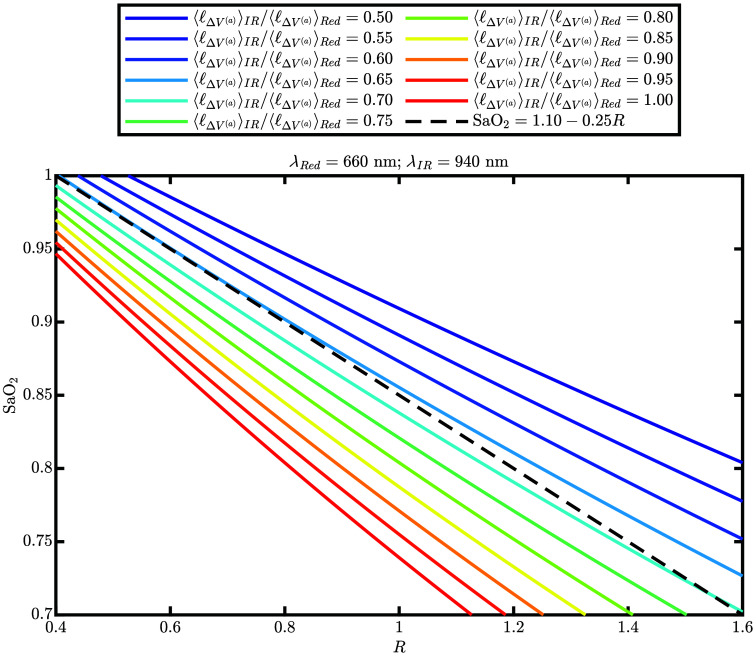
(Solid lines) Arterial oxygen saturation (${\mathrm{SaO}}_{2}$) versus ratio-of-ratios (R) according to Eq. (14) [i.e., derived from Beer–Lambert law (mBLL)] for various values of the amplitude ratio of the pulsatile mean partial optical path length through the arterial compartment at infrared (IR) and red wavelengths ($\langle \ell_{\Delta V^{\left(a\right)}}\rangle_{\mathrm{IR}}/\langle \ell_{\Delta V^{\left(a\right)}}\rangle_{\text{Red}}$). (Dashed line) ${\mathrm{SaO}}_{2}$ versus R according to the TIDA-00301 reference design equation. ^18^ All lines consider the optical wavelengths (λ’s) in the TIDA-00301 reference design so that $\lambda_{\text{Red}}=660\;\mathrm{nm}$ and $\lambda_{\mathrm{IR}}=940\;\mathrm{nm}$.

Equation (14) is the key result of this derivation because it shows the relationship between ${\mathrm{SaO}}_{2}$ and R and importantly explains how the coefficients relate to ε’s and $\left\langle \ell_{\Delta V^{\left(a\right)}}\right\rangle$’s. Figure 1 plots ${\mathrm{SaO}}_{2}$ versus R according to Eq. (14) for various values of ${\left\langle \ell_{\Delta V^{\left(a\right)}}\right\rangle }_{\mathrm{IR}}/\langle \ell_{\Delta V^{\left(a\right)}}\rangle_{\text{Red}}$, considering known hemoglobin extinction coefficients (ε’s)^19^ for the optical wavelengths (λ’s) of 660 and 940 nm.

### 2.1 Assuming a Linear Relationship Between Arterial Oxygen Saturation (${\mathrm{SaO}}_{2}$) and Ratio-of-Ratios (R)

Although Eq. (14) is indeed used in pulse oximetry,^16^ it can also be linearized. This is common in pulse oximetry, where a linear relationship between ${\mathrm{SaO}}_{2}$ and R is assumed. To linearize Eq. (14), we use the Taylor expansion

$${\mathrm{SaO}}_{2}\approx {\frac{A+BR_{0}}{C+DR_{0}}+\frac{\partial {\mathrm{SaO}}_{2}}{\partial R}|}_{R=R_{0}}(R-R_{0}),$$ (19)

where

$${\frac{\partial {\mathrm{SaO}}_{2}}{\partial R}|}_{R=R_{0}}=\frac{BC-AD}{(C+DR_{0}{)}^{2}}.$$ (20)

For the sake of example, let $R_{0}=1$, which corresponds to a range of ${\mathrm{SaO}}_{2}$ values around 0.85. Linearizing about other values of R may be done to increase the accuracy of the resulting linear equation about a certain range of ${\mathrm{SaO}}_{2}$; for example, if we assume ${\left\langle \ell_{\Delta V^{\left(a\right)}}\right\rangle }_{\mathrm{IR}}/{\left\langle \ell_{\Delta V^{\left(a\right)}}\right\rangle }_{\text{Red}}=0.65$, linearizing about $R_{0}=0.4$ would result in maximum accuracy about ${\mathrm{SaO}}_{2}=1.0$ (Fig. 1). The range of typical values of R in literature depends on various factors, most notably the choice of wavelengths. However, to give an example range of values, R may vary between ∼0.4 and 1.4 when we focus on the range of 1.0 to 0.7 for ${\mathrm{SaO}}_{2}$ using 660 and 940 nm.^18^ For $R_{0}=1$, the Taylor expansion leads to the following linearized relationship:

$${\mathrm{SaO}}_{2}\approx \frac{A+B}{C+D}+(\frac{BC-AD}{(C+D{)}^{2}})(R-1),$$ (21)

which we can write as

$${\mathrm{SaO}}_{2}\approx \alpha +\beta R,$$ (22)

where

$$\alpha =\frac{AC+2AD+BD}{(C+D{)}^{2}},$$ (23)

$$\beta =\frac{BC-AD}{(C+D{)}^{2}}.$$ (24)

Here, it is important to remember the definitions of A, B, C, and D [Eqs. (15)–(18)], which just depend on ε’s and $\left\langle \ell_{\Delta V^{\left(a\right)}}\right\rangle$’s.

All ε’s are known^19^ (given knowledge of the wavelengths), and only ${\left\langle \ell_{\Delta V^{\left(a\right)}}\right\rangle }_{\mathrm{IR}}/\langle \ell_{\Delta V^{\left(a\right)}}\rangle_{\text{Red}}$ is unknown. For this example, we take the same λ’s as in the Texas Instruments TIDA-00301 reference design^18^ so that $\lambda_{\text{Red}}=660\;\mathrm{nm}$ and $\lambda_{\mathrm{IR}}=940\;\mathrm{nm}$. If we assume ${\left\langle \ell_{\Delta V^{\left(a\right)}}\right\rangle }_{\mathrm{IR}}/{\left\langle \ell_{\Delta V^{\left(a\right)}}\right\rangle }_{\text{Red}}=0.65$, then α=1.08 and β=−0.23, so that Eq. (22) becomes

$${\mathrm{SaO}}_{2}\approx 1.08-0.23R,$$ (25)

which compares closely to the equation in the TIDA-00301 reference design,^18^ which is

$${\mathrm{SaO}}_{2}\approx 1.10-0.25R.$$ (26)

Aside from the TIDA-00301 reference design, this equation is also widely used in the pulse oximetry literature^20^^–^^23^ and is in good agreement with another reported linearly calibrated equation from the literature that does not apply the assumption to remove the natural logarithm in Eqs. (8) and (9)^17^

$${\mathrm{SaO}}_{2}\approx 1.138-0.2487R.$$ (27)

Figure 1 visualizes the non-linearized version [Eq. (14)] for these wavelengths (i.e., 660 and 940 nm) and also plots the TIDA-00301 reference design^18^ equation [Eq. (26)] for comparison. This comparison shows that the TIDA-00301 reference design equation corresponds to a $\langle \ell_{\Delta V^{\left(a\right)}}\rangle_{\mathrm{IR}}/\langle \ell_{\Delta V^{\left(a\right)}}\rangle_{\text{Red}}$ value of ∼0.65 for $1\leq {\mathrm{SaO}}_{2}\leq 0.9$ and ∼0.70 for $0.75\leq {\mathrm{SaO}}_{2}\leq 0.70$. This suggests that the ratio $\langle \ell_{\Delta V^{\left(a\right)}}\rangle_{\mathrm{IR}}/\langle \ell_{\Delta V^{\left(a\right)}}\rangle_{\text{Red}}$ ranges from ∼0.65 to 0.70 when ${\mathrm{SaO}}_{2}$ decreases from 1.0 to 0.7. This is a much smaller range of $\langle \ell_{\Delta V^{\left(a\right)}}\rangle_{\mathrm{IR}}/\langle \ell_{\Delta V^{\left(a\right)}}\rangle_{\text{Red}}$ values than the one considered in Fig. 1 (0.50 to 1.00), which is the approximate range associated with the same ${\mathrm{SaO}}_{2}$ decrease from 1.0 to 0.7 in an ideal case of a homogeneous increase in the concentration of arterial blood in the tissue.

## 3 Discussion

As can be seen from Fig. 1 and the numerical example in Eq. (25), the TIDA-00301 reference design equation ^18^ is a linear approximation of Eq. (14) associated with a fixed value for $\langle \ell_{\Delta V^{\left(a\right)}}\rangle_{\mathrm{IR}}/\langle \ell_{\Delta V^{\left(a\right)}}\rangle_{\text{Red}}$ of ∼0.65. When calibration is carried out for such pulse oximetry equations, all ε’s and $\langle \ell_{\Delta V^{\left(a\right)}}\rangle_{\mathrm{IR}}/\langle \ell_{\Delta V^{\left(a\right)}}\rangle_{\text{Red}}$ are empirically estimated. We caution thinking of a dichotomy between NIRS (i.e., mBLL-derived methods) and pulse oximetry as the underlying physics is the same.

There are a few implicit assumptions in the derivation we would like to discuss. The first arises in Eq. (5), where it is assumed that all measured pulsatile changes result from blood-volume changes in the arteries alone (i.e., $\Delta V^{\left(a\right)}$ is the only source of intensity change ΔI). The ubiquity and success of pulse oximetry suggest that this assumption is reasonable. Built into this assumption is that the pulsatile oscillations of $\Delta [{\mathrm{HbO}}_{2}]$ and Δ[Hb] in the probed tissue volume are in phase with each other. In fact, contributions from pulsatile blood flow would result in out-of-phase oscillations of $\Delta [{\mathrm{HbO}}_{2}]$ and Δ[Hb].^24^ As a matter of fact, some work has shown not fully in-phase pulsatile oscillations of $\Delta [{\mathrm{HbO}}_{2}]$ and Δ[Hb],^24^^,^^25^ leading us to suggest further investigation of this assumption.

The second implicit assumption we would like to discuss is the introduction of $\left\langle \ell_{\Delta V^{\left(a\right)}}\right\rangle$ in Eqs. (8) and (9). In general, optical path lengths must be introduced when using mBLL, but their meaning can be nuanced. This is one of those cases. As we isolate only pulsatile oscillations in pulse oximetry analysis, we are assuming that ΔI only comes from the vascular compartment that is associated with the pulsatile oscillation. Therefore, Eqs. (8) and (9) were introduced, in which $\left\langle \ell_{\Delta V^{\left(a\right)}}\right\rangle$ is the average partial optical path length in the pulsatile arterial blood volume (i.e., in $\Delta V^{\left(a\right)}$). Thus, $\left\langle \ell_{\Delta V^{\left(a\right)}}\right\rangle$ is difficult to know, and we know of no definitive way to measure it even with FD or TD NIRS. This is because knowledge of $\left\langle \ell_{\Delta V^{\left(a\right)}}\right\rangle$ requires knowing two things: first, the spatial distribution of absolute optical properties in the tissue, and second, the spatial distribution of pulsatile arteries. This is likely different for every person who undergoes a pulse oximetry measurement; thus, we posit that the success of pulse oximetry (with calibration that needs to be constant across people) is relying on the fact that only the ratio of $\left\langle \ell_{\Delta V^{\left(a\right)}}\right\rangle$’s at different wavelengths appears in Eqs. (14) and (22). With this in mind, we also suggest further investigation of this question of the variability of $\left\langle \ell_{\Delta V^{\left(a\right)}}\right\rangle$ (or the ratio of $\left\langle \ell_{\Delta V^{\left(a\right)}}\right\rangle$’s) across different subjects. This is a possible physical explanation of the racial disparities already observed in pulse oximetry methods^26^^,^^27^ or possible biases from blood content or finger size. Finally, we observe that $\langle \ell_{\Delta V^{\left(a\right)}}\rangle_{\mathrm{IR}}/\langle \ell_{\Delta V^{\left(a\right)}}\rangle_{\text{Red}}$ is not strictly constant because it depends on ${\mathrm{SaO}}_{2}$, which affects the distribution of absolute optical properties in tissue. This is related to a known issue where the pulse oximetry calibration curve becomes inaccurate at low values of ${\mathrm{SaO}}_{2}$,^28^ for which calibration adjustments as a function of ${\mathrm{SaO}}_{2}$ have been proposed.^29^ The analysis presented in this work [leading to Eq. (14)] shows that the dependence of $\langle \ell_{\Delta V^{\left(a\right)}}\rangle_{\mathrm{IR}}/\langle \ell_{\Delta V^{\left(a\right)}}\rangle_{\text{Red}}$ on ${\mathrm{SaO}}_{2}$ is the key factor to consider for adjusting the calibration curve as a function of ${\mathrm{SaO}}_{2}$. Figure 1 suggests that the dependence of $\langle \ell_{\Delta V^{\left(a\right)}}\rangle_{\mathrm{IR}}/\langle \ell_{\Delta V^{\left(a\right)}}\rangle_{\text{Red}}$ on ${\mathrm{SaO}}_{2}$ is weaker than in an ideal case of a homogeneous increase of arterial blood volume, and more research is needed to quantify such dependence in conditions relevant to pulse oximetry. We observe that $\left\langle \ell_{\Delta V^{\left(a\right)}}\right\rangle$ also depends on the source-detector geometry of the pulse oximeter. Source-detector geometry is different in transmittance and reflectance-based pulse oximetry. Therefore, $\langle \ell_{\Delta V^{\left(a\right)}}\rangle_{\mathrm{IR}}/\langle \ell_{\Delta V^{\left(a\right)}}\rangle_{\text{Red}}$ may feature a different sensitivity to confounders in reflectance versus transmittance geometries. This suggests that investigations on how $\langle \ell_{\Delta V^{\left(a\right)}}\rangle_{\mathrm{IR}}/\langle \ell_{\Delta V^{\left(a\right)}}\rangle_{\text{Red}}$ is influenced by different collection geometries may be fruitful.

Finally, we would like to discuss the matter of the hemoglobin extinction coefficients (ε’s), especially related to the spectral features of the sources. First, let us assume that we are using laser diodes (LDs) and know the wavelength of both the red and IR sources. In this case, both Eqs. (14) and (22) have two unknowns. This requires noticing that Eq. (15) for A and Eq. (17) for C used in Eq. (14) only depend on ε’s, which are known if source wavelengths are known^19^ albeit with some uncertainty.^30^ For this reason, we see no real advantage to Eq. (22) if we have knowledge of the source wavelengths as both Eqs. (14) and (22) have two unknowns, but Eq. (22) introduces more assumptions. Furthermore, we could go one step further to claim that Eq. (14) really only has one unknown, which is $\langle \ell_{\Delta V^{\left(a\right)}}\rangle_{\mathrm{IR}}/\langle \ell_{\Delta V^{\left(a\right)}}\rangle_{\text{Red}}$, which may simplify calibration even further. Now, we consider that most pulse oximeter devices use light-emitting diodes (LEDs), not LDs. In fact, the broad spectral features of these sources have been suggested as a reason for the observed skin-tone bias.^31^^,^^32^ In the case of LEDs, the discussion of calibration and ε above is still valid. However, ε’s would be replaced with a weighted average (⟨ε⟩), weighted by the LED emission spectrum,^16^ which requires calibrating the LED spectrum. With all these considerations in mind, we see a great advantage in reconsidering the use of LDs in pulse oximeter devices as the advantage may outweigh the cost and safety concerns (which could be mitigated by adding a diffuse material to the LD).

## 4 Conclusion

The difference between conventional pulse oximetry and NIRS methods based on the mBLL lies in the consideration of $\varepsilon_{{\mathrm{HbO}}_{2}}$, $\varepsilon_{\mathrm{Hb}}$, and $\left\langle \ell_{\Delta V^{\left(a\right)}}\right\rangle$ by the latter as opposed to empirical calibration or linearity assumptions between ${\mathrm{SaO}}_{2}$ and R, by the former. In this perspective, we have leveraged the common underlying physics of diffuse optics to link the empirical approach of pulse oximetry and the analytical description of the mBLL. The key equations are Eqs. (14) and (22), with the former coming directly from the mBLL and the latter being a linear approximation of Eq. (14). Noting that both equations have two unknowns [A and C are known in Eq. (14), albeit with some uncertainty, as they are only related to extinction coefficients], we see little advantage to Eq. (22). With this in mind, we suggest that more emphasis be placed on calibrating to Eq. (14) in pulse oximetry, but we acknowledge that this would require knowledge of the source wavelengths. The value of Eq. (14) is also that it specifies the physical meaning of the four coefficients (i.e., A, B, C, and D), thus providing specific indications on the impact of various error sources. We hope that this exercise of deriving ${\mathrm{SpO}}_{2}$ from mBLL is informative and helpful to other researchers in both the fields of NIRS and pulse oximetry.

## Data Availability

Applicable supporting code and data are available from the authors upon reasonable request.
